# Durable Management of Plant Viruses: Insights into Host Resistance and Tolerance Mechanisms

**DOI:** 10.3390/biology15020205

**Published:** 2026-01-22

**Authors:** Muhammad Zeshan Ahmed, Chenchen Zhao, Calum Wilson, Meixue Zhou

**Affiliations:** Tasmanian Institute of Agriculture, University of Tasmania, Launceston 7250, Australia; muhammadzeshan.ahmed@utas.edu.au (M.Z.A.); chenchen.zhao@utas.edu.au (C.Z.); calum.wilson@utas.edu.au (C.W.)

**Keywords:** plant–virus interactions, zig-zag model, resistance (*R*) genes, RNA interference (RNAi), plant defense signaling pathways, breeding for virus resistance

## Abstract

Plant viruses cause substantial yield and quality losses worldwide, and their rapid evolution can erode deployed host resistance. This review synthesizes current knowledge of antiviral resistance and tolerance mechanisms, using barley yellow dwarf virus (BYDV) in cereals as an illustrative case study. We first summarize key layers of plant antiviral immunity, including pre-formed physical and chemical barriers, dominant and recessive resistance genes, RNA silencing, hormone-regulated defense signaling, and degradation pathways such as the ubiquitin–proteasome system and selective autophagy. We then discuss how these mechanisms are exploited in breeding and biotechnology, covering conventional introgression, marker-assisted selection, QTL mapping and pyramiding, induced variation (mutation breeding and TILLING/ecoTILLING), transgenic strategies (pathogen-derived resistance and plantibodies), RNA interference-based approaches, and CRISPR-enabled editing of susceptibility factors. Finally, we highlight emerging nano-enabled tools and propose integrated strategies that combine genetic resistance with surveillance and vector management to improve durability under climate change and ongoing viral diversification.

## Simple Summary

Plant viruses pose a serious threat to food security and greatly reduce agricultural productivity worldwide. Plants rely on complex defense mechanisms to combat viral infections, and both transgenic and non-transgenic strategies are employed in resistance breeding. Modern technologies offer new ways to disrupt virus–plant compatibility. A deeper understanding of these systems supports the sustainable development of crops with durable resistance to viral diseases.

## 1. Plant Viruses: Major Constraint to Crop Production

Plant viral diseases represent one of the major biotic constraints to global agriculture, causing significant yield and quality losses across an extensive scale of crops. Plant viruses are small-genome, obligatory intracellular parasites that rely on plant machinery for replication and for intracellular (cell-to-cell) and systemic movement [1,2]. After gaining control of the host cell, the virus rapidly replicates and produces viral proteins [3]. Virions or viral ribonucleoprotein complexes can then move through the vascular system from the site of infection to distant tissues, causing characteristic disease symptoms such chlorosis, leaf curling, and stunted development [4,5]. Climate change is expected to intensify viral disease pressure by altering the distribution, abundance and activity of insect vectors, including aphids and whiteflies [6]. Although estimating global losses is difficult, the economic impact of plant viruses is commonly estimated to exceed $60 billion annually [7]. Genetic resistance remains among the most efficient and sustainable strategies for managing plant viral diseases and underpins many breeding programs. However, the frequent occurrence of new viral strains driven by high mutation rates, recombination, and pseudo-recombination can overcome existing resistance, and resistance genes can impose selection that favors resistance-breaking strains [8]. Barley yellow dwarf virus (BYDV), a persistently transmitted aphid-borne virus complex affecting cereals, exemplifies these challenges and is used here as a case study to discuss durable resistance and tolerance strategies.

## 2. Mechanism of Plant Resistance Against Viruses

This section summarizes major antiviral resistance and tolerance mechanisms, including pre-formed barriers, *R* gene-mediated responses, RNA silencing, hormone-regulated signaling, and degradation pathways such as the ubiquitin–proteasome system and selective autophagy.

### 2.1. Physical and Chemical Barriers

The plant’s primary defense against viral infection is made up of chemical and physical barriers. Structural features such as thicker cuticles, wax layers, leaf trichomes, and modified epidermal tissues can impede virus entry by limiting vector probing or mechanical penetration [9,10]. In parallel, changes in chemical cues such as surface metabolites, secondary compounds, or volatile organic compounds can influence insect vector attraction and feeding behavior [11], with documented effects on virus transmission efficiency. When plant viruses overcome preformed barriers such as cuticle thickness or vector deterrents, their presence may still be recognized by the plant. This recognition can activate cellular defense responses such as hypersensitive reactions, callose deposition, and reactive oxygen species production that restrict or slow further viral replication and movement within the host [12].

### 2.2. Dominant Resistance R Gene-Mediated Defense Responses

Plants have evolved two evolutionarily interrelated mechanisms to detect and recognize invading microbes. These systems work in a coordinated manner to mount effective defenses against a broad range of pathogens, including viruses. The dynamic interaction among hosts and pathogens has been conceptualized by (2006) through the zig-zag model, which illustrates the sequential and co-evolutionary “arms race” between plant defense mechanisms and pathogen attack strategies [13]. Unlike bacteria and fungi, viruses generally do not produce extracellular pathogen-associated molecular patterns (PAMPs) that can be identified by plant pattern recognition receptors (PRRs), such as receptor-like proteins (RLPs) and receptor-like kinases (RLKs) [14,15]. Because viruses rely on the host cell for all aspects of their replication, resistance models established for fungi and bacteria do not fully apply. As primary defense factors, PRRs trigger the first layer of resistance in other pathogens [16] and are largely ineffective against viruses due to the absence of extracellular PAMPs. Instead, virus perception occurs primarily through intracellular mechanisms, including RNA silencing pathways and *R* gene-mediated recognition. Nevertheless, this recognition can activate defense responses analogous to PAMP-triggered immunity (PTI), such as calcium influx, mitogen-activated protein kinase (MAPK) activation, production of reactive oxygen species (ROS), salicylic acid signaling, nitric oxide synthesis, callose deposition, and hypersensitive response (HR) [17]. By contrast, extracellular recognition mechanisms remain highly relevant in the context of virus vectors, where plants can detect vector-associated cues and activate protective responses [18].

According to modified zig-zag model proposed by (2014), RNA silencing is regarded as a central antiviral mechanism of PTI. Viral RNA silencing suppressors (RSSs) act as effectors that counteract host silencing machinery, thereby undermining PTI [19]. In turn, these RSSs can themselves be recognized by the host immune system as virulence factors, which triggers effector-triggered immunity (ETI) [19]. Several viruses, such as Rice stripe virus (RSV), Cauliflower mosaic virus (CaMV), and Plum pox virus (PPV) encode such suppressors to overcome PTI [20].

ETI provides a second layer of defense, triggered when specific viral effectors are recognized by corresponding intracellular resistance (R) proteins encoded by host *R* genes. An effective ETI halts pathogen progression, whereas a weak ETI allows disease establishment and susceptibility. ETI is often associated with a hypersensitive response (HR), a localized form of programmed cell death (PCD) at the site of infection, which produces necrotic lesions in otherwise healthy tissue. This localized cell death limits the virus to the infection site and prevents its spread into surrounding tissues. While PTI involves recognition of conserved patterns and generally does not involve HR, ETI is characterized by this rapid cell death response as the first visible phenotype of *R* gene mediated resistance [13].

Recognition in ETI is highly specific: when a viral effector protein is detected by an *R* protein, it is referred to as an avirulence (*Avr*) protein because it renders the pathogen non-virulent. In plants lacking the cognate *R* gene, however, the same effector can enhance virulence by suppressing PTI or interfering with host cellular processes. Viral *Avr* proteins, like those of other pathogens, may also have structural or enzymatic functions that facilitate infection [21].

Wheat (*Triticum aestivum* L.) and barley (*Hordeum vulgare* L.) have been researched for resistance to BYDV-PAV [22]. Several genes for BYDV-PAV tolerance were identified in wheat (*Bdv1*, *Bdv2*, *Bdv3* and *Bdv4*) [23] and barley (*Ryd1* [24] *Ryd2* [25] *Ryd3* [26] and *Ryd4* [27]. The successfully introduced genes for breeding resistant varieties include *Ryd2* in barley varieties (such as Atlas68, Wysor, Wbon, and Travira) and *Bdv2* in wheat varieties (such as Mackellar and Glover), which have demonstrated high levels of resistance to BYDV-PAV. These resistance phenotypes correspond to a low virus titer, a decrease in green grain weight per spike (GRS-R), and a low visual symptom score (VSS) [22]. A summary of various *R* genes and their functions is summarized in Table 1.

### 2.3. Recessive R Gene Resistance

Recessive *R* genes have also been identified in addition to dominant *R* genes, and the majority of these provide viral resistance [48]. The recessive *R* genes are plant genes which are necessary for the virus to finish its life cycle. These genes frequently controlled resistance by an incompatible viral–host relation, in which the virus causes infection, but host resistance mechanisms prevent further systematic infection. Eukaryotic translation initiation factors, eIF4E and eIF4G, are crucial for effective infection in several diseases which are caused by viruses such as Carmoviruses, Cucumoviruses, Potyviruses, Ipomoviruses, Begmoviruses, Sobemoviruses, and Waikiviruses. Many crop species are effectively protected against potyvirus infection by the natural variation in eIF4E. As a result, many plants’ natural resistance genes that serve as crucial host factors for virus infection have been identified and are being used in genome editing techniques to create plant virus resistance.

In many crops, genetic variation in eIF4E naturally provides efficient resistance to potyvirus infection. Many host factors that influence viral susceptibility have been recorded. These genes are utilized by using genome-editing technologies to generate plants with improved virus resistance.

Recognition of viral pathogens through dominant or recessive resistance genes initiates a cascade of defense responses, but the ultimate outcome is strongly shaped by plant hormones. These hormones act as regulators of defense signaling rather than as independent resistance mechanisms. Among them, jasmonic acid (JA), salicylic acid (SA), and ethylene (ET) are the most prominent participants.

Plant hormones function as central components of intercellular and systemic signaling networks, where they not only regulate developmental processes but also orchestrate responses to diverse biotic and abiotic stresses [49].

In the context of viral infections, SA is generally linked with antiviral resistance through the establishment of systemic acquired resistance (SAR) and the reinforcement of hypersensitive response (HR)-associated signaling. The function of SA in viral defense was first demonstrated in the classic interaction between the TMV and the tobacco *N* resistance gene [50]. Relying on virus–plant interaction, SA can impact three major phases of the virus disease cycle within the diseased host: intercellular movement, systemic movement, and viral replication [51]. Extensive research on SA-mediated plant defense has shown that SA is needed for the development of both local and systemic acquired resistance (SAR) in addition to stimulating a hypersensitive response (HR). SA stimulation produces an enhances the transcription of pathogenesis-related (*PR*) genes and promotes ROS production along with callose deposition during viral infection [51].

In contrast to systemic acquired resistance (SAR) induced by salicylic acid (SA), Jasmonic acid (JA), along with ethylene (ET), generally regulates induced systemic resistance (ISR), another significant systemic defense response in plants [52]. While SA is traditionally associated with defense against biotrophic pathogens, JA is more commonly linked with defense against necrotrophic pathogens. However, the function of Jasmonic acid in host–virus interactions is complex and somewhat ambiguous. Certain studies suggest that inhibition of the JA reaction may be crucial for successful geminivirus diseases, as JA has been shown to play a protective role during compatible interactions. For instance, prolonged JA administration reduced the DNA titer of beet curly top virus [53]. Interestingly, new research has also connected JA with the RNA silencing pathway, where JA-mediated host resistance is found after viral infection via microRNAs. Furthermore, studies with a tenuivirus, rice stripe virus (RSV), have demonstrated that the viral coat protein triggers JA biosynthesis and signaling, leading to the upregulation of JA-induced transcription factors that initiate the host defense network mediated by ARGONAUTE 18 (AGO18) [54]. Treating the *A. thaliana* plants with exogenous jasmonate causes disruption of geminivirus infection, showing that the suppression of the jasmonate response might be essential for successful for infection [53].

Brassinosteroids (BRs) are steroid plant hormones that regulate numerous parameters of plant growth and development [55]. BRs also contribute to immunity by activating host defenses against viral pathogens [56]. Levels of key BR biosynthesis intermediates, such as castasterone and deoxocastasterone, increased in TMV-infected tobacco leaves. Brassinolide (BL) application on tobacco increased the resistance against TMV; however, this resistance was not linked with salicylic acid deposition or the induction of pathogenesis-related (*PR*) genes, suggesting that BL-mediated resistance works autonomously of systemic acquired resistance (SAR) [56]. Additionally, studies have indicated that BR signaling was critical for the induction of tolerance against CMV [57].

Ethylene (ET) plays a dual role in virus-induced symptom development and systemic resistance. ET is produced upon viral infection and relates to symptom appearance. Increased ET production is related to lesion development and symptom severity in TMV-infected plants [58]. ET perception is also required for the establishment of SAR in TMV-infected leaves, which consequently triggers high SA concentration and SAR development in non-infected leaves [59]. In tomato plants, treatment with *Trichoderma harzianum* strain T-22 resulted in systemic resistance against CMV infection through the coordinated action of JA/ET and SA signaling pathways [60].

On the other hand, overexpression of NtERF5, an ET-responsive transcription factor of *N. tabacum*, increased transgenically produced tobacco’s resistance to TMV [61]. Plant responses to various biotic and abiotic stressors are significantly influenced by abscisic acid (ABA). Tomatoes having the *Tm-1* gene, which enables defense against tobacco mosaic virus, were shown to have greater quantities of abscisic acid compared to susceptible plants [62]. Exogenous administrations of ABA have been shown to inhibit the systemic accumulation of TMV-cg, a strain of TMV. Mutations in genes associated with ABA production and signaling, such as ABA deficient ab1, ab2, ab3, or abi4 in Arabidopsis resulted in rapid systemic accumulation of TMV-cg [63]. By blocking the transcription of β-1,3-glucanase, ABA also contributes to the rise in callose deposition on plasmodesmata, which may limit the virus’s ability to migrate from cell to cell and promote resistance [64].

Additionally, auxin, cytokinin, and gibberellin signaling pathways may be disrupted by viral infections. The replicase protein of TMV interacts with Aux/IAA related proteins in tomato and Arabidopsis, leading to changes in auxin-mediated gene regulation and disease development [65]. Gibberellic acid (GA) may also play a defensive function against biotrophic or necrotrophic pathogens by modulating the balance between SA and JA/ET-mediated signaling pathways [51].

It is quite clear that plant hormones are essential signaling molecules that regulate numerous functions in plants, including growth, development, and responses to biotic and abiotic stresses. Viral infections can disrupt the balance of phytohormones in plants. Changes in hormone levels have been associated with the development of symptoms and the accumulation of viruses within plant tissues. There is a correlation between the symptoms exhibited by virus-infected plants and alterations in phytohormone levels. These changes in hormone levels can influence the severity of symptoms and the extent of viral replication within the plant. Significant progress has been made in understanding how plant hormones, such as salicylic acid (SA), jasmonic acid (JA), ethylene (ET), and others, contribute to plant defense against viral infections. These hormones can induce defense responses that inhibit viral replication and systemic movement. Despite advancements in our understanding of hormone-mediated antiviral defenses, there are still many unanswered questions. One such question is how different hormone pathways interact and communicate with each other to regulate defense response against viral diseases. Understanding the crosstalk between hormone pathways is essential for elucidating the complex mechanisms underlying plant–virus interactions and developing more effective strategies for virus control in crops.

### 2.4. Atypical Dominant Viral Resistance Proteins (ADVRPs)

Over the past ten years, research on viral resistance has also led to the identification of certain dominant resistance genes that work independently as conventional innate immune signaling pathways. The proteins produced by these dominant resistance genes differ structurally from one another and from the ordinary *R* proteins and so cannot be incorporated into the standard plant innate immunity framework. Evidence indicates that many of these dominant resistance genes presumably work by directly interacting with viral proteins to suppress their function. After this, we referred to the proteins of this dominant resistance like Atypical Dominant Viral Resistance Proteins (*ADVRPs*). Prominent members of *ADVRP* family include lectins, carbohydrate-binding proteins with other catalytic domains; for example, glycosidase, glucanase, kinase, and ribosome-inactivating protein (RIP). Notable lectin-like *ADVRPs* include Restricted TEV Movement 1 (RTM1) and Jacalin-Type Lectin Required for Potexvirus Resistance1 (JAX1).

RTM1 mediates resistance to potyviruses, including Tobacco etch virus (TEV) and Lettuce mosaic virus (LMV), by disrupting the systemic movement of viral particles or ribonucleoprotein complexes in the phloem. Unlike traditional resistance mechanisms, RTM1-mediated resistance is independent of hypersensitive response (HR), salicylic acid (SA), or systemic acquired resistance (SAR) [66].

JAX1, another lectin-like *ADVRP* of Arabidopsis gives broad-spectrum resistance to potexviruses by identification of viral RNA-dependent RNA polymerase (RdRp) and blocking its function [67].

As research grows more advanced, the repertoire of *ADVRPs* will keep expanding. The source and evolutionary history of these *ADVRPs* is still unknown, though. It is plausible that these proteins have non-antiviral original roles and have acquired the capacity to limit virus multiplication by accident [68]. For example, yeast Nedd4 family E3 ubiquitin ligase Rsp5p that ordinarily plays a role in endocytosis [69] may bind to replication proteins (p33 and p92) of tomato bushy stunt virus (TBSV) and facilitates its destruction [70]. The resistance that some *ADVRPs* mediate, however, could potentially be a conserved antiviral mechanism. For example, since their discovery, lectins have been linked to antiviral activity [71].

### 2.5. Translational Repression Mechanisms-Based Resistance

To prevent viral replication without causing cell death, several *R* genes activate translational repression mechanisms. Besides HR, a different resistance mechanism occurs in Potato Virus X disease, in which Rx-1 provides extreme resistance (ER) via limiting viral multiplication while avoiding necrotic cell death [30]. There have been several attempts to identify Rx-1’s downstream signaling pathway. According to research, Ran GTPase-Activating protein 2 is a crucial interaction partner that aids in locating cytoplasm-situated Rx-1 CNL to the nucleus following its perception of Avr from PVX [72]. Researchers have discovered Golden-like (NbGLK1), a new transcription factor from *N. benthamiana* that, after interacting with viral CP106, brings nuclear-localized Rx-1 to the GLK binding site and stimulates transcription of the genes involved in extreme resistance [73]. The execution of distinct defensive responses to PVX by means of hypersensitive response or extreme resistance is influenced by both the nucleocytoplasmic trafficking of Rx-1 and the concentration of CP [74]. Interestingly, *N* gene of *N. benthamiana*, which encodes a TIR-NBS-LRR protein, can also induce translational arrest of PVX transcript in *N. benthamiana*, by using an Argonaute-mediated repression mechanism, which is different from the Rx-1-mediated pathway [75]. A further example of extreme resistance is seen in *Rsv3* gene-mediated response in soybean cultivar L29 against the avirulent strain of Soybean mosaic virus (SMV) G5H. In this case, extreme resistance is achieved via fast stimulation of autophagy and siRNA-mediated pathways rather than programmed cell death and reactive oxygen species buildup, ultimately clearing the infection [76].

### 2.6. Protein Degradation-Mediated Resistance

The ubiquitin–proteasome system (UPS) plays an important function in plant antiviral defense by targeting viral proteins for degradation, limiting their replication and spread. Ubiquitination, the process that drives this system, involves a multistep enzymatic cascade: ubiquitin (Ub) is activated by E1 enzymes, transferred to E2 enzymes, and finally conjugated to target proteins with the help of E3 ligases, marking them for degradation by the 26S proteasome [77]. This mechanism ensures cellular protein homeostasis and participates in nearly all plant antiviral defenses.

When viruses infect plants, their harmful proteins are tagged with ubiquitin, leading to their breakdown in the proteasome. This not only prevents viral replication but can also trigger immune responses by exposing viral fragments to the plant’s immune system. E3 ligases, which largely determine the specificity of ubiquitination, enable plants to target both endogenous and exogenous (viral) proteins. For example, Arabidopsis encodes over 1400 E3 ligases, reflecting their critical role in defense [78].

Specific E3 ligases have been identified for targeting viral proteins. The *Nicotiana tabacum* Ring Finger Protein 1 (NtRFP1), a RING-type E3 ligase, mediates the degradation of the βC1 protein from the tomato yellow leaf curl China virus (TYLCCNV), suppressing its proliferation [79]. Similarly, ubiquitination counteracts the replication-associated protein (RdRp) of turnip yellow mosaic virus (TYMV) in Arabidopsis [80]. Additionally, Cell-Division-Cycle protein 48, a conserved chaperone, facilitates the degradation of the movement protein (MP) of tobacco mosaic virus by extracting it from the endoplasmic reticulum to the cytosol [81].

### 2.7. Autophagy Pathways to Target Viral Proteins

Autophagy is a conserved mechanism that delivers unnecessary or aberrantly folded protein clusters as well as malfunctioning organelles to the vacuole for degradation and recycling. This is a very important process in plants for retaining cellular homeostasis under regular circumstances and surviving with both abiotic and biotic stressors [82]. Autophagy is connected to nearly every facet of cell physiological functions, including plant immunology, just as is ubiquitination [83]. Autophagy has a variety of roles in plant immunity, including maintaining HR and maintaining the homeostasis of immunity-signaling components [84]. In general, when a plant cell is infected by a virus, various stress signals are generated because of the infection. These stress signals can trigger the initiation of autophagy. Autophagy begins with the formation of autophagosomes, which are double-membraned structures, and these autophagosomes engulf cellular components or cargo that need to be degraded, which can include viral components. During a virus infection, autophagosomes can selectively sequester viral components. This sequestration may be a result of the cell recognizing viral proteins or nucleic acids as cargo to be removed. Autophagosomes then fuse with lysosomes, which are cellular organelles containing enzymes that can break down the contents of autophagosomes. Once fusion occurs, the viral components within the autophagosomes are broken down by lysosomal enzymes. This helps in eliminating the virus from the infected cell. The process of autophagy can also be linked to immune signaling in plants. It can contribute to the stimulation of defense responses and the production of antimicrobial compounds.

Plants also employ selective autophagy to break down protein aggregates encoded by viruses, ribonucleoprotein, and even whole viral particles, demonstrating that autophagy operates as different antiviral mechanisms. The significance of this route in slowing the proliferation of viruses has been emphasized in several recent reports; Autophagy-Related Gene 6 which is also named as BECLIN1, the key component of autophagy, interacts with Nuclear Inclusion Protein B, the RdRp of Turnip Mosaic Virus, to suppress viral replication [85].

Autophagy-Related Gene (*ATG8*) precisely interacts with the βC1 of cotton leaf curl Multan virus (CLCuMuV)-associated betasatellite, promoting its degradation and thereby inhibiting viral replication [86].

The autophagy cargo receptor NEIGHBOR OF BRCA1 (NBR1) interacts with particles of Cauliflower mosaic virus and unassembled CP to regulate their autophagy-dependent destruction, hence reducing the development of viral infection [87]. Additionally, by targeting HcPro, which is likely linked to virus-induced RNA granules, NBR1 inhibits TuMV accumulation.

## 3. Breeding and Genetic Approaches for Developing Virus Resistance in Plants

This section reviews how antiviral mechanisms are translated into crop improvement, from conventional breeding and marker-assisted selection to QTL mapping, gene pyramiding, induced variation (mutation breeding and TILLING), and transgenic and genome-editing strategies.

### 3.1. Breeding Approaches

#### 3.1.1. Traditional Breeding Approach

Traditional breeding for the developing pathogen-resistant cultivars may be traced back over a century, at a time when the inheritance patterns of specific resistance (*R*) genes and their associated effector-encoding genes were not yet identified at the molecular level. However, the fundamental principles of inheritance established by Mendel in the late 1800s were already known. The earliest document of such a breeding endeavor was in 1905, crossing and finding for disease-resistant English wheat [88]. Backcrossing technique was first used in crop breeding in 1922 by [89]. *R*-gene breeding quickly adopted a recurring backcrossing strategy to transmit necessary alleles from donors to commercially significant cultivars. The introgression of a disease-resistance trait into an elite background frequently needs at least six backcrosses [90], depending on the lines being crossed. There are several instances of disease-resistant cultivars developed by classical *R*-gene breeding. Traditional breeding has been the mainstay for developing viral pathogen-resistant cultivars in many crops. In wheat (*Triticum aestivum*), resistance against barley yellow dwarf virus-PAV (BYDV-PAV) has been achieved through the introgression of tolerance genes such as *Bdv1*, *Bdv2*, *Bdv3*, and *Bdv4*, with cultivars like Mackellar and Glover demonstrating high levels of resistance [22,23]. Similarly, in barley (*Hordeum vulgare*), BYDV-PAV resistance has been conferred through genes *Ryd1*, *Ryd2*, *Ryd3*, and *Ryd4*, with *Ryd2* successfully incorporated into varieties such as Atlas68, Wysor, Wbon, and Travira, resulting in low symptom severity, reduced virus titers, and minimal yield loss [24,25,26,27]. In tomato (*S. lycopersicum*), resistance to tomato yellow leaf curl virus (TYLCV) has been obtained by introgressing Ty-1, Ty-2, and Ty-3 from wild relatives such as *S. habrochaites and S. chilense*, leading to cultivars like TY172 and TY180 that show durable resistance under field conditions [91]. These examples illustrate that traditional breeding, including the identification of resistant germplasm, gene introgression, and selection for agronomic performance, continues to contribute a critical part in managing viral diseases in crops worldwide. For decades, genetic-mediated resistance techniques consisted of harnessing natural variability of plants by introgression of resistance genes by process of conventional breeding. However, the problem of crop resistance durability was brought up by the emergence of evolved pathogens that could defeat the resistance, often very quickly after resistance deployment. This triggered the need to create novel breeding techniques. Recent years have witnessed the rise in innovative ways of combining modern technological advances and cutting-edge scientific knowledge on plant–virus interactions, driving crop-yield enhancement and initiating a new era of agriculture innovation.

#### 3.1.2. Marker-Assisted Selection (MAS)

The backcrossing program eventually used marker-assisted selection, which significantly sped up the breeding process [92]. By applying DNA-marker technology, many breeding constraints can be circumvented. Leading breeding organizations now regularly utilize molecular markers closely associated with *R* genes in marker-assisted selection (MAS) programs to quickly and effectively transfer *R* genes and other beneficial features into elite crop-breeding lines. Nonetheless, DNA markers generated from the *R* gene sequence are the most effective. Without the need for challenging and time-consuming phenotypic screenings, markers enable each *R* gene to be precisely tracked during each breeding generation. Consequently, additional plant generations each year can be performed. MAS can ease the simultaneous introduction (i.e., ‘pyramiding’) of numerous *R* genes targeting distinct races of the same pathogen into one elite breeding line.

#### 3.1.3. Resistance Associated QTLs

Quantitative resistance is naturally controlled by multiple genes associated with specific genomic regions, known as quantitative trait loci (QTLs), each contributing to the overall resistance phenotype to varying degrees. While over 80% of characterized plant-virus resistances are controlled by a single gene, most agronomic traits in crops are polygenic and display quantitative inheritance, rather than segregating as simple, single-gene (monogenic) traits. Mapping QTL for quantitative resistance needs large-sized progenies, almost-saturated genetic maps, as well as accurate and quantitative phenotyping methodologies. As of now, comparatively few QTLs studies have been undertaken in plant–virus interactions relative to other diseases [93]. It is a conventional belief that resistance tends to be more durable when a virus requires higher number of mutations necessary to overcome it.

In barley, sources of natural resistance to BYDV, apart from the *Ryd2*, *Ryd3*, and *Ryd4Hb* genes, are also present. In a group of doubled haploid (DH) lines obtained from the Post × Vixen cross and several QTLs for PAV-BYDV, resistances were found on 2HL, 3HL (at a region encoding the *Ryd2* gene), 4H, and 7H chromosomes [94]. Likewise, QTLs for resistance to BYDV-PAV and -MAV serotypes were found on 1H, 4H and 7H in a group of 94 DH lines obtained by cross of Shyri × Galena [95]. Two QTLs were found in both Tasmanian experiments [96]. QTL Qbyd-3H correlates to the known resistance gene *Ryd2* [96], based on the location of markers associated with *Ryd2* [97]. On chromosome 5H, another QTL known as *Qbyd-5H* was regularly found. Three genes (*HORVU5Hr1G096560*, *HORVU5Hr1G096620*, and *HORVU5Hr1G096630*) have been functionally annotated as disease-resistance genes, making them potential candidates for *Qbyd-5H* [96]. Other potential genes within the area include *HORVU5Hr1G099940* and *HORVU5Hr1G102250*; both encode disease resistance protein [96]. Remarkably, the vernalization gene *Vrn-H1* (*HORVU5Hr1G095630*), which encodes a MADS-box transcription factor, is also located within the interval of Qbyd-5H [96]. This suggests a potential relationship between vernalization and disease resistance in barley. Two putative QTLs, named *Qbyd-7Ha* and *Qbyd-7Hb*, were identified on chromosome 7H. These QTLs were specifically detected in the Western Australia trials, where symptoms of BYDV infection were more severe. Although *Qbyd-7Ha* and *Qbyd-7Hb* contributed relatively less to the total phenotypic variation than *Qbyd-3H* and *Qbyd-5H*, they were still significantly associated with BYDV resistance. *Qbyd-7Hb* overlaps with previously reported associated QTLs which exhibit resistance to BYDV strains (MAV, PAV) [96]. The genomic intervals of *Qbyd-7Ha* and *Qbyd-7Hb* include 28 and 41 genes, respectively. Additionally, two disease-resistance genes (*HORVU7Hr1G114850* and *HORVU7Hr1G114880*) have positions within the interval of *Qbyd-7Hb*, suggesting their possible relevance in BYDV resistance [96].

Analogously to barley, multiple QTLs for BYDV-PAV resistance have been identified in wheat; for example, 22 QTLs in the Opata × Synthetic population and seven QTLs in the Frontana × INIA 66 population. Importantly, one of them matched the genetic location of the *Bdv1* gene on chromosome 7DS [23]. A QTL on chromosome 5A at roughly 230 cM was discovered [98]. In addition, a QTL on chromosome 6A approximately 5 cM was discovered in the DH population [98].

#### 3.1.4. Resistance-Gene Pyramiding

Resistance-gene pyramiding can broaden resistance spectra and improve durability, because the virus must accumulate multiple, simultaneous resistance-breaking mutations to overcome stacked loci [99]. For BYDV, pyramiding could combine tolerance genes such as *Bdv2* and *Bdv4* in wheat, or h *Ryd2* and *Ryd3* and cereals yellow dwarf virus (CYDV) resistance QTLs in barley. Stacking and major-effect loci may reduce viral replication and mitigate the effects of more aggressive isolates. However, pyramiding also presents several challenges. The approach is time consuming [100], and relatively little information exists regarding commercial cultivars developed using this method [101]. Pyramiding numerous genes or QTLs requires diverse strategies, including multiple-parent or complex crosses, backcrossing, and recurrent selection [101]. Molecular markers can facilitate effective marker-assisted selection, backcrossing, and pyramiding approaches [99].

The selection of appropriate genes for stacking is critical. Ideally, genes conferring resistance to different pathogen variants should be combined. For example, *Th. ponticum*/*intermedium* recombinant translocations, known as the Pontin series, contain *Bdv2*, *Sr25*, and *Lr19*, conferring resistance to BYDV, stem rust, and leaf rust, respectively [102].

Despite these benefits, *R*-gene pyramiding carries risks. One concern is that stacking *R* genes could select for “super pathogens” that overcome all pyramided genes, potentially leading to severe crop losses. Another challenge is determining the most suitable resistance genes for stacking across diverse growing regions [103]. Integrating differential gene stacking with complementary strategies, such as crop rotation or partial resistance deployment, can help mitigate these risks while maintaining durable, heritable resistance.

#### 3.1.5. Mutation Breeding (Induced Variation)

Mutation breeding induces novel genetic variation by exposing plant seeds or tissues to physical (e.g., gamma rays) or chemical mutagens (e.g., EMS), creating mutants with improved virus resistance. This approach is especially valuable where natural resistance is absent in the gene pool [104]. Plants are screened for virus resistance among the mutated progenies. Mutation breeding in mung bean produced lines with enhanced resistance to *Mungbean yellow mosaic virus* [105]. The integration of mutation breeding with molecular screening enables identification of point mutations in resistance-related loci, offering a non-transgenic and stable approach for durable virus resistance.

#### 3.1.6. TILLING and Eco TILLING

TILLING (Targeting Induced Local Lesions in Genomes) is a technique used to identify mutations in specific genes of interest. It involves classical mutagenesis, in which plant seeds or tissues are treated with chemical mutagens, usually ethyl methanesulfonate (EMS), to randomly introduce point mutations throughout the genome. Targeted mutation screening is then performed to identify mutagenized plants carrying alterations in the gene of interest, which can be exploited for breeding purposes [106]. By producing artificial polymorphism directly into crops allows potentially (i) the replacement of resistance alleles that have been overcome by evolving viral strains, (ii) generation of a novel resistance variant with broader spectrum, and (iii) to create entirely new resistance gene based on knowledge from heterologous systems. In doing so, TILLING expands natural allelic diversity through the identification of new, artificially derived alleles. The primary benefit of TILLING is its broad applicability; it can be applicable to all diverse plant species, irrespective of its genome size, ploidy level, or mode of propagation. Importantly, it does not involve the introduction of heterologous DNA, unlike genetically modified (GM) approaches, even though this strategy necessitates the prior characterization of the gene conferring the resistance. Similarly, a natural variant of the TILLING approach (using natural germplasm collections instead of EMS mutant’s collections) is named ecoTILLING and consists of harnessing the complete natural diversity of plant species including wild-related and cultivated genotypes [107]. TILLING and ecoTILLING, which were first established in *A. thaliana*, have quickly expanded to other model plants (false-brome, barrelclover, Japanese lotus) and significant crops (e.g., *Zea mays*, *Glycine max*, *Sorghum bicolor*, *Solanum lycopersicum*, *Capsicum annuum*, *Pisum sativum*, *Triticum aestivum*, *Musa acuminata*, *Oryza sativa*, *Hordeum vulgare*, *Phaseolus vulgaris*, *Cucumis sativus*, *Brassica napus*). With particularly effective instances of recent applications to antiviral defense, it is now evident that these approaches are becoming important crop-improvement tools [108].

### 3.2. Transgenic Approaches

#### 3.2.1. Host-Derived or Cross-Species Transgenic Approach

A broad class of transgenic approaches leverages the plant’s own resistance machinery or modifies it to reduce compatibility with viruses. These strategies include transferring or overexpressing canonical resistance (*R*) or nucleotide-binding leucine-rich repeat (*NLR*) genes across genetic backgrounds.

*NLR*/*R*-gene transfer across genetic backgrounds, or intra-family gene transfer has been frequently successful owing to the conservation of immune signaling components within plant families. A classic example is the *Rx* gene from potato, which encodes a CC-NLR protein that confers high level of potato virus X (PVX) resistance. The *Rx* protein mediates resistance in both potato and Nicotiana species, demonstrating efficacy across Solanaceae backgrounds. Another example is the *N* gene from tobacco, which confers resistance to tobacco mosaic virus (TMV) and retains functionality in related Nicotiana species. Rx- or N-mediated resistance arrests viral infection and suppresses virus accumulation while causing minimal downstream pathology under many conditions.

By contrast, transferring *R*/*NLR* genes across distant taxonomic groups (for example, between plant families) often gives weak or no resistance because the recipient may lack compatible downstream signaling components (helper NLRs, chaperones, signaling cofactors). This limitation is sometimes referred to as restricted taxonomic functionality (RTF). For instance, a sensor NLRs that functions in Solanaceae may require a specific helper NLR network that is absent in more distantly related species, so transferring the sensor alone may not reconstitute immunity. Recent studies show that co-transfer of matched sensor and helper NLRs can overcome RTF and extend immune receptor functionality between angiosperms [109]. Even with successful transfers, inappropriate expression (e.g., misregulated promoters, constitutive high expression) can cause autoimmunity or fitness penalties. To reduce vulnerability to virus escape, breeders often stack multiple *R* genes so that the virus would need to acquire multiple and compensatory mutations simultaneously to overcome the combined resistance.

#### 3.2.2. Pathogens Derive Resistance (PDR)

Plant viruses, with their small genomes encoding only a limited set of essential proteins for replication, movement, and encapsidation, have long been considered suitable targets for engineered resistance through the concept of pathogen-derived resistance (PDR), first proposed by [110]. In *PDR,* genes (or gene fragments) from the virus itself are inserted into the host genome to create resistance. The first successful approaches involved protein-based resistance, where plants were engineered to produce viral proteins (mainly coat proteins, CP) which conferred resistance in several RNA viruses such as, for example, TMV, AlMV, CMV, PVX, and TRV. The latter finding supported that the protection was not really because of the viral protein itself; instead, it was nucleic-acid-mediated resistance: the viral gene sequences introduced into the plant triggered the plant’s natural RNA-silencing machinery. RNA silencing breaks down matching viral RNA, stopping the infection. This was a major paradigm shift [111,112]. Modern strategies now employ hairpin RNAs, inverted repeat constructs, and artificial microRNAs (amiRNAs) to trigger the generation of virus-derived small interfering RNAs (siRNAs) that specifically target viral genomes for degradation [113,114]. These approaches have proven highly effective in conferring strong and often broad-spectrum resistance under both laboratory and field conditions.

A notable success story is the control of papaya ringspot virus in Hawaii through the deployment of transgenic papaya [115]. While concerns over biosafety and regulation continue to limit the widespread deployment of virus-resistant transgenic crops, RNA silencing-based resistance is now recognized as the predominant and most reliable transgenic strategy for combating plant viral diseases.

#### 3.2.3. Resistance by Means of Plantibodies

As a substitute for protein-mediated PDR or pre-activation of innate antiviral defense strategies like RNA silencing, biotechnological techniques provide the chance to use ways that are wholly unique to plants. An analogous strategy is the production of antibodies, extensively employed in animals to identify infections. Even though plants lack the defensive system related to these proteins, some antibodies’ affinities can be strong enough to impair crucial activities of a viral protein in plants. Though technically demanding, the synthesis of single-chain variable fragment (scFv) antibodies, the invention of the phage-display technique and the generation of synthetic scFv libraries have substantially increased the usability of this technology [116].

The first reported application of plantibodies demonstrated reduced susceptibility to artichoke mottle crinkle virus via an scFv targeting the coat protein [117]. However, broader adoption faced challenges, particularly the instability of scFvs in plant cells, as most viruses replicate in the cytoplasm. Strategies such as targeting scFvs to the endoplasmic reticulum (ER) using the ‘KDEL’ retention signal have stabilized scFvs and improved their efficacy [118]. This approach enabled successful expression of scFvs in plants against beet necrotic yellow vein virus [119] and resistance to tobacco mosaic virus [120].

Further studies expanded the application of scFvs to other viruses. For example, scFvs derived from a monoclonal antibody against johnsongrass mosaic virus reduced susceptibility to potato virus Y (PVY) and clover yellow vein virus [121]. Similarly, scFvs targeting non-structural proteins essential for virus replication, such as RNA-dependent RNA polymerase (RdRp), provided resistance to tomato bushy stunt virus and related viruses [122]. High expression of scFvs against PVY’s NIa protein offered complete protection [123]. Future advancements in antibody structure and alternative protein scaffolds may enhance scFv stability and overcome current limitations, broadening their applicability as a sustainable tool for plant virus resistance [124].

#### 3.2.4. RNA-Silencing-Based Strategies for Virus Resistance

RNA gene silencing, also termed RNA interference (RNAi), is a central basal antiviral defense in plants [125]. A classic field phenotype of RNAi is ‘recovery’, where previously symptomatic plants produce new leaves with reduced symptoms and virus titers [126].

During infection, double-stranded RNA (dsRNA) generated from viral replication intermediates or structured viral RNAs is processed by Dicer-like (DCL) enzymes into 21–24 nt small interfering RNAs (siRNAs). These siRNAs are loaded into Argonaute (AGO)-containing RNA-induced silencing complexes (RISC), which guide sequence-specific cleavage or translational repression of complementary viral RNAs [127,128,129]. The delay creates a “cellular battleground”, where silencing competes with viral suppressor proteins. Silencing predominates in healthier tissues, leading to reduced symptoms or recovery, whereas suppression allows severe disease [127,130]. Like innate immunity, RNA silencing perceives dsRNA in a sequence-independent manner, amplifies signals, and can spread systemically via virus-derived siRNA duplexes [130]. Unlike pattern-triggered immunity (PTI), it does not involve classical defense outputs such as PR-gene induction, hypersensitive response, or signaling molecules like salicylic acid, ROS, or calcium. Recent studies suggest that dsRNAs may function as viral PAMPs [131], with PTI components implicated in their recognition [132], though the mechanism remains unresolved.

Understanding RNAi mechanisms has enabled development of highly effective antiviral strategies. Plants can be pre-programmed to activate RNA silencing against viral sequences, tipping the balance in favor of the host before viral suppressors are expressed [125,133]. Several RNAi-based strategies have been applied for crop protection. Hairpin RNA (hpRNA) constructs containing inverted viral repeats trigger systemic and durable virus resistance. Artificial microRNAs (amiRNAs), engineered from native miRNA precursors, allow precise targeting of viral sequences and multiplexing to overcome viral diversity [134]. Trans-acting siRNA (tasiRNA) systems amplify silencing signals and broaden protection. Early sense/antisense constructs demonstrated RNA-based resistance but have largely been replaced by hpRNA and amiRNA designs.

Virus-induced gene silencing (VIGS) vectors can transiently silence viral or host susceptibility genes but are mostly limited to research due to biosafety concerns. Non-transgenic alternatives such as host-induced gene silencing (HIGS) and spray-induced gene silencing (SIGS) apply dsRNA via host expression or topical delivery, offering environmentally friendly, GMO-free protection. For example, clay nanosheet-based dsRNA sprays provided prolonged antiviral defense [135], and transgenic papaya resistant to papaya ringspot virus represents a successful field application [136]. Future perspectives include multiplexed RNAi designs targeting conserved viral genes (e.g., polymerase, replicase), integration with gene editing of susceptibility genes, and advanced dsRNA formulations for stable, large-scale field use.

#### 3.2.5. CRISPR-Cas-Mediated Gene Editing Resistance

Conventional breeding played a pivotal role in enhancing plant resistance to pathogens by developing and screening large populations over multiple generations; however, this approach is labor intensive and time consuming [137]. In recent decades, genome engineering approaches, including Zinc Finger Nucleases (ZFNs), Transcription Activator-Like Effector Nucleases (TALENs), and CRISPR/Cas9, have advanced the modification of plant immune components, enabling long-term resistance against pathogens [138]. CRISPR/Cas9 is particularly attractive because it is cost-effective, time-efficient, and precise, overcoming many limitations of ZFNs and TALENs such as high cost, complexity, and off-target effects [139,140].

CRISPR/Cas9 has been exploited to develop plant protection against both DNA and RNA viruses. RNA viruses exploit plant host elements towards continuing their life cycle. To continue their life cycle, RNA viruses depend on host components such eukaryotic translation initiation factors (eIF4E, eIF(iso)4E, and eIF4G) [141]. CRISPR/Cas9 targeting of these genes has produced encouraging outcomes. Chandrasekaran effectively induced mutation inside the two distinct locations of the cucumber (*Cucumis sativus*) translation initiation factor eIF4E gene by CRISPR-Cas9 resulting in the Cucumis eIF4E mutant [142]. Resistance to cucumber vein yellowing virus (CVYV), zucchini yellow mosaic virus (ZYMV), and papaya ringspot mosaic virus-W (PRSV-W) was found when homozygous Cucumis mutant plants were tested against a Potyviridae family member. In further investigation, isoform of *eIF4E* gene locus was targeted in *Arabidopsis thaliana* to produce a 1-base-pair precise mutation inside the gene [143]. Genetically modified plants demonstrated complete resistance to Potyvirus and Turnip Mosaic Virus (TuMV) without showing any significant off-target effects.

Additionally, CRISPR technology has also been employed for the control of RNA viruses. FnCas9 expressed and targeted TMV and CMV by employing specific sgRNAs in Arabidopsis and *N. benthamiana* [144].

Researchers have documented a 40–80% reduction in virus accumulation, and stable sgRNA-FnCas9-mediated resistance against tobacco mosaic virus (TMV) and cucumber mosaic virus (CMV) up to the T6 generation, demonstrating heritable resistance. This finding also uncovered that it is RNA-binding activity rather than endonuclease activity of FnCas9 which is responsible for interference with the CMV genome. This mechanism helps to restrict the escape of mutated viral variants from host resistance. Cas13a was employed to target Turnip Mosaic Virus (TuMV) RNA, focusing on key viral genes, including coat protein (CP) and helper component-proteinase (HC-Pro) [145]. Their approach reduced TuMV replication and spread in tobacco plants.

CRISPR/Cas9 has also been used to develop resistance against Rice Tungro Spherical Virus (RTSV) in rice (*Oryza sativa*). Mutations were induced in the *eIF4G* gene using three gRNAs at specific SNP positions [146]. By advancing progenies to the T2 and T3 generations via Agrobacterium-mediated transformation, they successfully generated RTSV-resistant IR-64 rice lines. Taken together, the above-reported studies demonstrate the effectiveness of CRISPR/Cas9 in creating disease-resistant crops. However, challenges such as off-target effects and potential virus escape mechanisms remain. Future research should focus on refining CRISPR/Cas9 design and identifying additional susceptibility (S) genes across diverse plant species. This will pave the way for sustainable and precise genetic engineering solutions to plant diseases.

## 4. Nano-Based Viral Management

This section highlights nano-enabled approaches for viral disease management, including antiviral nanomaterials and the use of nanoparticles to deliver protective molecules or improve diagnostics, and discusses opportunities and constraints for field application.

The study of creating, modifying, and using materials and technologies at the molecular, atomic, or supramolecular level is known as nanotechnology [147]. In the realm of plant protection, nanomaterials can be employed as a protectant or as a carrier for precise and targeted administration by conjugation with pesticide active ingredients, adsorption, or encapsulation. Nanomaterials can be applied as a foliar spray for seed treatment and root application due to their recognized antifungal, antibacterial, and antiviral properties [148]. Nonmetals, metalloids, metallic oxides, and carbon nanomaterials are among the NPs showing plant-disease-suppressive activities [149]. Copper, zinc oxide, gold, titanium oxide, iron, and especially silver are the most frequently investigated and employed NPs [150]. Many phytopathogenic viruses, including Bean Yellow Mosaic Virus (BYMV) [151], Cucumber Mosaic Virus (CMV) [152], Potato Virus Y (PVY) [153], and others, have been discovered to be susceptible to the NPs. Direct contact with virus particles, preventing their adsorption or entrance into host cells, and obstructing their multiplication or systemic transit throughout the plant are some of the suggested antiviral actions of nanoparticles. Beyond direct suppression, nanoparticles can also operate as elicitors of plant defense responses or serve as sensitive diagnostic instruments in the form of nano-biosensors for early viral identification.

## 5. Discussion: Comparative Evaluation of Virus-Resistance Strategies

Conventional breeding has historically been the cornerstone of developing virus-resistant cultivars in crops such as wheat and barley [24,26,95]. This strategy leverages natural genetic variation, introgression of resistance (*R*) genes, and quantitative trait loci (QTLs) to achieve durable resistance [10,22]. Marker-assisted selection (MAS) has accelerated this process by enabling precise incorporation of resistance loci [92,102]. Despite these advantages, conventional breeding remains time consuming and often requires multiple generations to combine multiple *R* genes for effective protection. High viral mutation rates can also rapidly overcome single-gene resistance, underscoring the importance of gene pyramiding and continual screening [99,100]. Furthermore, climate change may exacerbate viral pressures, affecting host resistance and crop productivity [6].

Transgenic strategies, including pathogen-derived resistance (PDR), RNA interference (RNAi), and plantibodies, provide targeted and often rapid antiviral solutions [110,111,112,113,116,119]. RNAi-mediated silencing allows pre-activation of antiviral defenses and can overcome viral suppressors of RNA silencing [125,126,127]. Recombinant antibodies expressed in plants confer additional specificity, as demonstrated against PVY, ToMV, and ClYVV [116,117,122]. These methods can provide broad-spectrum and rapid resistance compared to conventional breeding. However, they face challenges such as regulatory constraints, biosafety concerns, and the potential emergence of resistance-breaking viral strains [8].

Genome editing technologies, particularly CRISPR/Cas systems, have emerged as precise tools to engineer antiviral resistance [137,142]. By targeting host susceptibility genes (e.g., eIF4E) or viral sequences directly, CRISPR/Cas9 or Cas13 can confer durable and heritable resistance without introducing foreign DNA [142,146]. These approaches have successfully generated resistance in multiple crops, including cucumber, Arabidopsis, and rice, and allow multiplex editing of several viral determinants simultaneously [142,143,144]. Nevertheless, concerns remain regarding off-target effects, viral evolution to bypass edits, and technical limitations in certain crop species [141].

Durability of resistance is a major consideration, especially given the high mutation rates and adaptive potential of plant viruses [8,66]. Pyramiding multiple *R* genes, combining RNAi with genome editing, or integrating conventional breeding with biotechnological approaches can substantially reduce the risk of resistance breakdown [91]. Understanding host–virus interactions, including the roles of NLR proteins, RNA silencing, ubiquitination, and phytohormone signaling, is critical for designing robust strategies [75,103,138].

Future directions should emphasize integrated approaches that combine conventional, transgenic, and genome editing strategies, informed by virus surveillance and bioinformatics to monitor emerging strains [2,3]. Multiplex genome editing, targeted pyramiding of resistance genes, and consideration of environmental factors such as global warming are key to achieving sustainable and durable virus resistance in crops [6,28].

## 6. Conclusions

Plant viral diseases continue to pose serious threats to crop productivity, food security, and sustainable agriculture. Advances in plant–virus biology have enabled the development of diverse resistance strategies, including conventional breeding, transgenic technologies, RNAi-based approaches, and CRISPR/Cas genome editing. Each strategy offers distinct advantages: conventional breeding provides stable and heritable resistance but is slow; transgenic and RNAi-based methods enable rapid and often broad-spectrum protection but face regulatory and biosafety challenges; genome editing delivers precise and potentially durable resistance, although issues such as off-target effects and viral evolution must be addressed.

Future efforts should focus on integrated resistance strategies that combine multiple approaches to enhance durability under high viral mutation pressure. The use of high-throughput sequencing, bioinformatics, and virus monitoring will be critical for early detection of emerging strains and for developing region-specific resistance solutions. Moreover, climate change is likely to alter host–virus interactions, emphasizing the need to identify resistance sources that remain effective across diverse environmental conditions.

Overall, the integration of conventional breeding, biotechnological tools, and genome-editing technologies guided by genomic insights and environmental considerations represents a sustainable pathway for developing virus-resistant crops. Such comprehensive approaches will be essential for protecting agricultural productivity, strengthening food security, and addressing future challenges in global agriculture.

## Figures and Tables

**Table 1:** *R* genes: a reliable source of resistance against viruses.

Virus	Family and Genetic Material	*R* Gene	Source	Avr	Type	Resistance Mechanism	References
Tobacco mosaic virus (TMV)	Tobamovirus, +strand RNA	*N*	Tobacco	p50 (TMV helicase domain)	TIR-NBS-LRR	The ATP/N complex binds to p50, which promotes ATP hydrolysis and initiates downstream signaling that results in a hypersensitive response.	[28]
Tomato mosaic virus (ToMV)	Tobamovirus, +strand RNA	*Tm-1*	False Turkey tail	Replication protein	CC-NBS-LRR	The TIM-barrel fold interacts with viral replication proteins, blocking the formation of the replication complex on host cell membranes.	[29]
		*Tm-2, 22*	Peruvian Tomato	Movement protein	CC-NBS-LRR	Inhibits viral movement through plasmodesmata	
Potato virus X (PVX)	Alphafexiviridae, +sense ssRNA	*Rx-1*	Andean Potato	Coat protein	CC-NBS-LRR	Provides extreme early-stage resistance, limiting virus accumulation without inducing a hypersensitive response.	[30]
		*Rx-2*	Wild Andean Potato	Movement protein	CC-NBS-LRR	The CC domains of Rx-1 and Rx-2 associate with RanGAP2 to trigger effector-mediated extreme resistance.	[31]
Tomato spotted wilt virus (TSWV)	Bunyaviridae, —strand ambisense RNA	*Sw-5b*, *6*, *7*, *1a*, *1b*	Peruvian Tomato	NSm (cell-to-cell movement protein)	SD-CC-NBS-LRR	Both SD and LRR domains recognize NSm as the Avr factor and trigger a hypersensitive response.	[32]
		*RTSW*	Sweet Tobacco	NSM	Not reported	It triggers HR-associated cell death and enhances the expression of defense-related genes. The elicitor region required for RTSW–NSm recognition is distinct from the site recognized by Sw-5b.	[33]
		*Tsw*	Habanero pepper	NSS (silencing suppressor)	CC-NBS-LRR	Activates a hypersensitive response that blocks systemic virus spread.	[34]
*Cucumber mosaic virus* (CMV)	Bromoviridae; ssRNA	*RCY1*	*Mouse ear cress*	Coat protein	CC-NBS-LRR	Activates salicylic acid and ethylene signaling pathways, conferring resistance.	[35]
Mung bean yellow mosaic virus (MYMIV)	Geminiviridae; DNA virus	*CYR1*	Mung bean	Coat protein	CC-NBS-LRR	The mechanism of resistance remains incompletely understood.	[36]
Soyabean mosaic virus (SMV)	Potyviridae; + sense ssRNA	*Rsv1*	Soyabean line (line PI 96983)	Hc-Pro, P3, CI	TIR-NBS-LRR	It has not been documented yet.	[37]
Soyabean mosaic virus (SMV)	Potyviridae +sense ssRNA	*Rsv3*	Soyabean line PI 96983	CI, P3	CC-NBS-LRR	It has not been documented yet.	
		*Rsv4*	Soyabean (PI 96983)	P3	CC-NBS-LRR	Provides extreme resistance by inhibiting viral replication while not triggering a hypersensitive response.	
		*Rsc4-3*	Soyabean (‘Dabaima’ cultivar)	CI	CC-NBS-LRR	The cell wall-localized Rsc4-3 detects viral proteins in the apoplast and triggers a hypersensitive response.	[38]
Tomato yellow leaf curl virus (TYLCV)	Geminiviridae, monopartite ssDNA	*Ty-2*	Wild tomato	Rep/C1	Not yet reported	Recognition of Rep/C1 induces a hypersensitive response.	[39]
Tomato leaf curl New Delhi virus (ToLCNDV)	Geminiviridae, Bipartite ssDNA	*Sw-5as*	Cherry tomato	AC4	SD-CC-NBS-LRR	The interaction between Sw-5a and the AC4 protein activates HR and promotes ROS production, restricting viral proliferation.	[40]
Potato virus Y (PVY)	Potyviridae; +sense ss RNA	*Rysto*	Wild potato	Coat protein	TIR-NBS-LRR	Depending on coat protein abundance and host factors, Rysto triggers either extreme resistance or a hypersensitive response.	[41]
Potato virus Y (PVY)	Potyviridae; +ss RNA	*Pvr4*	Sweet pepper	RNA-dependent RNA polymerase (NIb)	CC-NBS-LRR	Initiates a durable extreme-resistance response against the virus.	[42]
Pepper Mottle Virus (PepMoV)	Potyviridae, +ss RNA	*dominant resistance* *Pvr4, 7, 9*	Sweet pepper ‘CM334’, Hot chili pepper PI159236, Sweet pepper ‘CM334	RNA-dependent RNA polymerase (NIb)	CC-NBS-LRR	Confers both hypersensitive and extreme-resistance phenotypes.	[43]
Turnip crinkle virus (TCV)	Tombusviridae; RNA virus	*HRT*	*Mouse ear cress* Di-17	Coat Protein P38	CC-NBS-LRR	Induces HR-mediated cell death and activates expression of pathogenesis-related (PR) proteins.	[44]
Wheat streak mosaic virus (WSMV)	Potyviridae, monopartite positive-sense RNA	*Wsm1, 2, 3*	Wheat grass Tall wheat grass	Not yet reported	Not reported	Provides strong resistance by blocking long-distance virus movement at or below ~18 °C. At higher temperatures these *R* genes lose functionality.	[45]
Cassava mosaic geminiviruses (CMGs)	Geminiviridae, bipartite ssDNA	*Dominant monogenic resistance locus* *CMD2, 3*	Wild cassava Rubber Tree	Not yet reported	Not yet reported	It has not been documented yet.	[46]
Tobacco etches virus (TEV)	Potyviridae; +-sense ssRNA	*RTM1, 2*	Not reported	Not yet reported	Not yet reported	*RTM* genes activated in sieve elements and limit systemic movement.	[47]

Abbreviations: Cylindrical inclusion protein (CI); N requirement gene 1 (NRG1); viral non-structural protein (NSm); post-transcriptional gene silencing (PTGS); Solanaceae domain-coiled coil domain (SD-CC); and transcriptional gene silencing (TGS).

## Data Availability

No new data were created or analyzed in this study. Data sharing is not applicable to this article.
